# Overexpression of pigeonpea stress-induced cold and drought regulatory gene (*CcCDR*) confers drought, salt, and cold tolerance in *Arabidopsis*

**DOI:** 10.1093/jxb/eru224

**Published:** 2014-05-27

**Authors:** Srinath Tamirisa, Dashavantha Reddy Vudem, Venkateswara Rao Khareedu

**Affiliations:** Centre for Plant Molecular Biology, Osmania University, Hyderabad, 500 007, India

**Keywords:** Abiotic stress tolerance, *Cajanus cajan*, cDNA library, cold and drought regulatory gene, nuclear localization.

## Abstract

A potent cold and drought regulatory protein-encoding gene (*CcCDR*) was isolated from the subtractive cDNA library of pigeonpea plants subjected to drought stress*. CcCDR* was induced by different abiotic stress conditions in pigeonpea. Overexpression of CcCDR in *Arabidopsis thaliana* imparted enhanced tolerance against major abiotic stresses, namely drought, salinity, and low temperature, as evidenced by increased biomass, root length, and chlorophyll content. Transgenic plants also showed increased levels of antioxidant enzymes, proline, and reducing sugars under stress conditions. Furthermore, *CcCDR*-transgenic plants showed enhanced relative water content, osmotic potential, and cell membrane stability, as well as hypersensitivity to abscisic acid (ABA) as compared with control plants. Localization studies confirmed that CcCDR could enter the nucleus, as revealed by intense fluorescence, indicating its possible interaction with various nuclear proteins. Microarray analysis revealed that 1780 genes were up-regulated in *CcCDR*-transgenics compared with wild-type plants. Real-time PCR analysis on selected stress-responsive genes, involved in ABA-dependent and -independent signalling networks, revealed higher expression levels in transgenic plants, suggesting that CcCDR acts upstream of these genes. The overall results demonstrate the explicit role of CcCDR in conferring multiple abiotic stress tolerance at the whole-plant level. The multifunctional *CcCDR* seems promising as a prime candidate gene for enhancing abiotic stress tolerance in diverse plants.

## Summary

A potent multifunctional stress-induced cold and drought regulatory protein (CcCDR) has been isolated from pigeonpea. It conferred multiple abiotic stress tolerance through activation of ABA-dependent and ABA-independent genes in *Arabidopsis*.

## Introduction

Abiotic stress conditions such as drought, salinity, and extreme temperatures cause adverse effects on the overall growth and yield potential of diverse crop plants. Perception of stress signal(s) and activation of complex signalling pathway(s) bring about drastic changes in the cellular gene expression which is a prequisite for plants to acclimatize under extreme conditions (Kasuga *et al.*, 1999; Tong *et al.*, 2007). Although salt, drought, and cold stresses are different in nature, they are, however, known to activate certain sets of common genes in plants. Many stress-responsive genes, expressed in plants under stress conditions, have been analysed and their gene products are broadly classified into two groups. The first group includes different classes of proteins such as enzymes required for biosynthesis of various osmoprotectants, late embryogenesis-abundant (LEA) proteins, antifreeze proteins, chaperones, and detoxification enzymes, which directly protect cells. The second group includes signalling molecules, transcription factors, and protein kinases (Shinozaki and Yamaguchi-Shinozaki, 1997). Products of these genes also participate in the generation of regulatory molecules such as plant hormones, namely abscisic acid (ABA), ethylene, and salicylic acid (SA) (Xiong *et al.*, 2002). Among these, ABA is known to play a crucial role in the regulation of plant growth and development as well as modulating the plant response to various environmental factors (Xiong and Zhu, 2003; Nieva *et al.*, 2005).

Different transcription factors regulate target gene expression in several stress regulatory pathways―as evidenced by microarray analyses—indicating the occurrence of cross-talk between various stress signalling pathways (Fowler and Thomashow, 2002). Molecular and genomic analyses using model plants facilitated the resolution of complex networks and led to the discovery of additional mechanism(s) of stress tolerance (Umezawa *et al.*, 2006). By employing molecular biology tools and genetic approaches, several abiotic stress-inducible genes were isolated and their functions have been precisely characterized in transgenic plants (Park *et al.*, 2003 ). Several genes associated with abiotic stress tolerance have been transferred into different plants to improve their tolerance against specific stress conditions. Overexpression of the cold-induced plasma membrane protein gene (*MpRCI*) of plantain (*Musa paradisiac*) in tobacco resulted in increased tolerance to low temperature (Feng *et al.*, 2009). A NAC-type transcription factor (*OsNAC5*) of rice, when overexpressed in transgenic rice, caused increased tolerance to salinity (Takasaki *et al.*, 2010). Overexpression of *OsLEA3-1* in rice resulted in enhanced tolerance to drought under field conditions (Xiao *et al.*, 2007). Similarly, expression of *DREB1A* of *Arabidopsi*s, hybrid-proline-rich protein (*CcHyPRP*) and cyclophilin (*CcCYP*) genes of pigeonpea (*Cajanus cajan* L.), and *TaSnRK*2.8 and *TaSnRK*2.4 of wheat in *Arabidopsis* conferred tolerance to drought, salinity, and extreme temperatures (Mao *et al.*, 2009; Zhang *et al.*, 2010; Priyanka *et al.*, 2010; Sekhar *et al.*, 2010).

Pigeonpea (*C. cajan*) is a diploid legume crop grown mainly in semi-arid tropical regions. It has an excellent deep-root system with profuse laterals and grows well in hot and humid climates. Since it is one of the well known abiotic stress-tolerant food crops, it can be used as a source for identification of potential candidate genes in the improvement of stress tolerance of various crop plants (Priyanka *et al.*, 2010). In this study, a stress-responsive *C. cajan* cold and drought regulatory protein-encoding gene (*CcCDR*) was cloned. Overexpression of CcCDR in *Arabidopsis* afforded marked tolerance against drought, salinity, and cold stress conditions.

## Materials and methods

### Plant materials and treatments

Seeds of pigeonpea variety ICP 8744, obtained from the International Crops Research Institute for the Semi-Arid Tropics (ICRISAT), Hyderabad (India), were surface-sterilized with 0.1% (w/v) mercuric chloride for 5min, and washed thoroughly with sterile water. The washed seeds were germinated in Petri plates containing sterile wet blotting paper. Later, the germinated seedlings were transferred to pots and maintained in the greenhouse at 28±2 °C. To monitor the stress-inducible nature of the isolated gene, pigeonpea seedlings were subjected to polyethylene glycol (PEG)-6000 (20% w/v), NaCl (1.0M), and cold temperature (4 °C) for 6h.

Seeds of *A. thaliana* (ecotype Columbia) were treated with ethanol for 10min followed by 0.05% mercuric chloride for 3min, and were washed thoroughly six times with sterile water. The sterilized seeds, after stratification for 64h at 4 °C, were grown on Murashige and Skoog (MS; Murashige and Skoog, 1962) medium at 20±1 °C with a 16h photoperiod under fluorescent light (7000 lux at 20cm) in a Conviron growth chamber (Model TC16, Winnipeg, Manitoba, Canada).

### Construction of a subtractive cDNA library and isolation of the full-length *Cajanus cajan* cold and drought regulatory gene (*CcCDR*)

Total RNA was isolated from 4-week-old unstressed (–0.49±0.02 Mpa) and 20% PEG-treated (–1.01±0.02 Mpa) plants by the guanidinium thiocyanate method (Sambrook and Russell, 2001). Poly(A^+^) RNA was purified from total RNA by oligo(dT) cellulose chromatography using an mRNA isolation kit (Amersham Pharmacia Biotech, Asia Pacific Ltd, Quarry Bay, Hong Kong). The cDNA library was constructed using a PCR select cDNA subtractive hybridization kit (Clontech, Mountain View, CA, USA) employing 2 μg of mRNA of both control and PEG-treated plants. PCR products were ligated into the pGEM-T Easy vector (Promega Corporation, Madison, WI, USA) and transformed into *Escherichia coli* (Top10) cells (Sambrook and Russell, 2001). Cloned cDNA fragments were sequenced using an automated DNA sequencer.

### RACE PCR

Total RNA was isolated from root and leaf tissues of 4-week-old 20% PEG-stressed pigeonpea plants as described above, and cDNA was synthesized using a Clontech Marathon cDNA amplification kit. RACE (rapid amplification of cDNA ends) PCR was carried out employing primers 5′-GGATCTTGTCCTTCACCTTGTCCAT-3′ (gene specific) and 5′-CCATCCTAATACGACTCACTATAGGGC-3′ (adaptor specific) to extend the 5′ region of the *CcCDR* partial clone (accession no. GD173778). Amplified fragments were eluted and ligated into the pGEM-T Easy vector and transformed into *E. coli* cells, and recombinant clones were subjected to DNA sequencing. Homology search of sequences at the nucleotide and protein levels was carried out using BLAST (NCBI) and ExPASy tools. Multiple sequence alignment was performed employing CLUSTALW (www.genome.jp/tools/clustalw/). The full-length *CcCDR* (GU444042) coding sequence was amplified with *Pfu* DNA polymerase using 5′-GGGGATCCATGTCTGGGATCATCCACAAGA-3′ (forward, *Bam*HI site underlined) and 5′-GGGAGCTCTT AATCACTGTCGCTGCTGCTG-3′ (reverse, *Sac*I site underlined) primers.

### Southern blot analysis of pigeonpea plants

Genomic DNA (15 μg), isolated from pigeonpea plants by the cetyltrimethylammonium bromide (CTAB) method (Saghai-Maroof *et al.*, 1984), was digested independently with *Bam*HI, *Eco*RI, *Hin*dIII, and *Sal*I restriction enzymes. The digested DNA was resolved on an 0.8% agarose gel and was transferred to a positively charged nylon membrane as per the manufacturer’s instructions (Amersham Pharmacia Biotech). Southern blot analysis was performed according to Sambrook and Russell (2001). The *CcCDR* coding region (GU444042) was radiolabelled with [α-^32^P]CTP using Ready to Go DNA labelling beads (Amersham Pharmacia Biotech) and used as a probe.

### Northern blot analysis

Total RNAs (20 μg)―isolated from pigeonpea plants treated independently with PEG (20%), NaCl (1M), and cold (4 °C) for 6h along with untreated plants―were resolved on a denaturing agarose gel (1.5% agarose, 2.2M formaldehyde, and 1× MOPS). The separated RNAs were transferred to a positively charged nylon membrane, and northern blot was performed as described (Sambrook and Russell, 2001). The *CcCDR* coding region was radiolabelled as described above and used as a probe.

### Construction of plant expression cassettes and transformation of *A. thaliana*

The coding region (282bp) of *CcCDR* was cloned at the *Bam*HI and *Sac*I sites of the pBI121 vector under the control of either the *Cauliflower mosiac virus* (CaMV) 35S or the rd29A promoter. The pBI121 vector containing *CcCDR* and *npt*II expression units and the pBI121 vector with *npt*II (empty vector) were mobilized independently into the EHA105 strain of *Agrobacterium* through triparental mating. *Agrobacterium*-mediated floral dip transformation of *A. thaliana* was carried out using the vacuum infiltration method (Clough and Bent, 1998). Putative transformants were selected on MS medium supplemented with kanamycin (50mg l^–1^).

### Molecular analysis of transgenic *Arabidopsis* plants

PCR analysis was carried out using the genomic DNA isolated from kanamycin-tolerant plants. DNA from untransformed plants was used as a negative control, and plasmid DNA of pBI12135S*npt*II-35S*CcCDR*⁄pBI12135S*npt*II-rd29A *CcCDR* was used as a positive control. For PCR, plasmid DNA (10ng) and genomic DNA (50ng) were used as templates employing *CcCDR* gene-specific primers 5′-ATGTCTGGGATCATCCACAAGATT-3′ and 5′-TTAATCAC TGTCGCTGCTGCTGCT-3′. The reaction mixture, containing template, primers, buffer, dNTPs, and *Taq* DNA polymerase, was subjected to initial denaturation (94 °C) for 5min, followed by repeated denaturation (94 °C) for 45 s, annealing (60 °C) for 45 s, and elongation (72 °C) for 1min for a total of 35 cycles. The final elongation step was carried out at 72 °C for 10min. Amplified PCR products were analysed on a 1.0% agarose gel containing ethidium bromide. Genomic DNA (10 μg) of wild-type (WT) plants and transgenic lines was digested independently with *Hin*dIII and *Eco*RI restriction enzymes, and Southern blot was performed using the radiolabelled *CcCDR* coding region as described earlier.

### RT-PCR analysis

Total RNA was isolated independently from transgenic and control plants using the TRIZOL method (Invitrogen, Carlsbad, CA, USA). Real-time PCR (RT-PCR) was carried out using a reaction mixture containing TRIS-HCl (10mM), KCl (50mM), MgCl_2_ (1.5mM), dNTPs (200 μM each), *Taq* DNA polymerase (1.5U), MMLV reverse transcriptase (2U), total RNA (1.0 µg), forward primer (10 pM), and reverse primer (10 pM) of *CcCDR*. The reaction mixture (50 μl) was incubated at 50 °C for 30min and PCR was carried out as described earlier. Amplified PCR products were analysed on a 1.0% agarose gel.

### Construction of *GFP::CcCDR* fusion construct and transformation of *A. thaliana*

The coding sequence of *CcCDR* without the termination codon was fused with the 5′ region of green fluorescent protein (*GFP*) driven by the CaMV 35S promoter. The expression cassettes of CaMV35S–*CcCDR*::*GFP/*CaMV35S–*GFP* were subcloned into pCAMBIA 2301 containing the *hpt*II expression unit and mobilized into the EHA105 strain of *Agrobacterium* through triparental mating. Transformation of *A. thaliana* was carried out as described above. Transformed seedlings were selected on MS medium supplemented with kanamycin (50mg l^–1^). Callus was induced from the roots of CaMV35S–*GFP* and CaMV35S–*CcCDR*::*GFP* transgenic seedlings on MS medium containing 2,4-dichlorophenoxyacetic acid (2mg l^–1^). Individual cells derived from the callus were visualized under a confocal microscope (Leica Microsystems, Germany).

### Functional analysis of transgenics for abiotic stress tolerance

Seeds of WT, control (vector transformed or VT), and homozygous transgenic (T_3_) *Arabidopsis* CS1, CS2, RD1, and RD2 plants were surface-sterilized and placed on MS salt medium, and kept in the dark for 3 d at 4 °C for stratification. Later, they were transferred to a growth chamber (Conviron, TC16), and allowed to grow at 20±1 °C under long-day (16h light/8h dark cycles) conditions with fluorescent light (7000 lux at 20cm). To test for drought and salt tolerance, 2-week-old seedlings were grown on MS medium supplemented with mannitol (200mM) or NaCl (150mM) for 7 d. For cold tolerance studies, 2-week-old seedlings were transferred to an incubator set at 4 °C for 7 d. Later, the seedlings were transferred on to the MS salt medium and allowed to recover for 10 d under normal conditions (20±1 °C, 16h light/8h dark cycles, 7000 lux at 20cm) in the growth chamber. After 10 d of recovery, survival rate, root length, and biomass of seedlings were recorded.

### Relative water content measurements

Relative water contents (RWCs) of the 4-week-old CS1, CS2, RD1, and RD2 transgenic plants together with WT and VT plants were measured. Fresh weight loss was calculated relative to the initial plant weight. For fresh weight, plants were weighed immediately and left in the growth chamber (20±1 °C) until there was no further loss in weight (desiccated weight). Finally, plants were dried for 24h at 70 °C and dry weights were recorded. The RWCs of the samples were measured using the formula: RWC (%)=(desiccation weight–dry weight)/(fresh weight–dry weight)×100.

### Osmotic potential analysis

Two-week-old plants of each line were selected and frozen with liquid nitrogen for 30 s, and preserved at –80 °C. Sap from the leaves was collected by squeezing with the help of a micro pestle, and the osmotic potential of the sap was determined using a vapour pressure osmometer (Vapro, Model 5520; Wescor Inc., Logan, UT, USA).

### Cell membrane stability analysis

Cell membrane stability (CMS) was determined using a conductivity meter (Model Sension 5; HACH Company, Loveland, CO, USA). Ten-day-old seedlings grown on 1× MS medium were placed on filter paper saturated with NaCl (150mM) solution. As soon as symptoms of stress appeared (after 6h) in the WT and VT plants, seedlings were removed, rinsed thoroughly, and immersed in 20ml of double-distilled water at room temperature (20±1 °C). After a gap of 2h, the initial conductivity of samples was recorded. Later, samples were boiled for 30min, cooled to room temperature, and the final conductivity was measured. CMS was calculated using the formula: CMS (%)=1–initial electrical conductivity/electrical conductivity after boiling×100.

### Leaf chlorophyll content measurements

Leaf discs of CS1, CS2, RD1, and RD2 transgenic lines together with WT and VT plants were floated independently on water (control), 200mM mannitol, and 150mM NaCl solutions at room temperature (20±1 °C) and also on water at 4 °C for 72h. Leaf discs were then used for measuring chlorophyll content spectrophotometrically as described previously (Sekhar *et al.*, 2010).

### Measurement of proline content

Fifteen-day-old *CcCDR* CS1, CS2, RD1, and RD2 transgenic, WT, and VT seedlings were subjected to 200mM mannitol, 150mM NaCl, and 4 °C for 3 d. Plant tissues were homogenized in 3% aqueous sulphosalicylic acid and the homogenates were centrifuged at 1000rpm for 5min. Supernatant was collected and equal volumes of glacial acetic acid and ninhydrin were added. Quantification of proline was carried out as described by Bates *et al.* (1973).

### Estimation of reducing sugars

Samples of leaf tissues (100mg) were collected from the stressed (200mM mannitol/150mM NaCl/4 °C for 3 d) and unstressed transgenic, VT, and WT plants, frozen with liquid nitrogen and ground to powder. Reducing sugars were extracted from the powder twice with 80% ethanol at 95 °C. The supernatant collected was bulked and reduced to dryness at 80 °C for 2h. The residue was dissolved in 10ml of distilled water, and reducing sugar contents were estimated as described (Miller, 1959).

### Estimation of malondialdehyde (MDA)

For measurement of MDA, 50mg of tissue, collected from stressed and unstressed transgenic, WT, and VT seedlings, was homogenized with 2ml of 0.1% (w/v) cool trichloroacetic acid (TCA) on ice. The homogenates were centrifuged at 14 000 *g* for 10min at 4 °C, and then 250 μl of supernatant was mixed with 1.5ml of TCA/thiobarbituric acid (0.25% thiobarbituric acid containing 10% TCA) reagent. MDA content was estimated as per the method of Shin *et al.* (2012).

### Catalase assay

Fifteen-day-old *CcCDR* CS1, CS2, RD1, and RD2 transgenic lines together with WT and VT seedlings were subjected to 200mM mannitol, 150mM NaCl, and 4 °C for 3 d. Tissue was collected from seedlings grown under stress as well as unstressed conditions, homogenized with liquid nitrogen, and suspended in 0.1ml of 10mM PBS buffer (pH 7.0). Catalase activity was determined according to Shin *et al.* (2012).

### Superoxide dismutase (SOD) assay

Fifteen-day-old *CcCDR* CS1, CS2, RD1, and RD2 transgenic lines along with WT and VT seedlings were subjected to mannitol (200mM), NaCl (150mM), and cold (4 °C) for 3 d. Tissue collected from seedlings grown under stress as well as unstressed conditions was ground in liquid nitrogen and homogenized in solution containing 50mM KHPO_4_ (pH 7.0), 0.1mM EDTA, and 1% (w/v) polyvinylpolypyrrolidone. SOD activity was measured as described by Gupta *et al.* (1993).

### Detection of H_2_O_2_ and superoxide by diaminobenzidine (DAB) and nitroblue tetrazolium (NBT) staining methods

For visualization of H_2_O_2_ and superoxide generation as a result of abiotic stress, 2-week-old seedlings treated with mannitol (200mM), NaCl (150mM), and cold (4 °C) for 3 d, along with unstressed plants were employed for DAB and NBT staining. Stained samples were transferred to 80% ethanol and incubated at 70 °C for 10min to remove the chlorophyll. DAB staining was carried out as described by Du *et al.* (2008) for visualization of H_2_O_2_. The NBT staining method described by Wang *et al.* (2010) was used for superoxide detection

### Effect of ABA on seed germination and seedling growth

Seeds of transgenic and the WT plants were placed on MS medium containing different concentrations of ABA (0–2.0 μM) for recording the germination frequency. Further, germinated seedlings were allowed to grow for 20 d to assess their sensitivity to ABA.

### Measurement of stomatal aperture sizes

Rosette leaves of 3-week-old plants were detached and floated (abaxial side down) on a solution containing 10mM MES-KOH (pH 6.15), 30mM KCl, and 1mM CaCl_2_, and were incubated under light for 2h. Later, they were treated with ABA-containing solution for 2h. In each treatment, sizes of 50 stomatal apertures were measured under a confocal microscope.

### Microarray analysis

Two-week-old WT and transgenic (RD2) plants were subjected to mannitol (200mM) stress for 3 d. Total RNA was extracted from seedlings using TRIZOL reagent (Invitrogen). The quality and quantity of RNA were checked by Agilent Bioanalyzer. Labelled complementary RNA was generated using Agilent’s Quick-Amp labelling Kit (p/n5190-0442). Microarray analysis was performed using the Affymetrix Gene Chip 4X44K according to the manufacturer’s instructions (Affymetrix). Microarray data were normalized using the RMA algorithm implemented in GeneSpring software. Analysis of the data was performed using GeneSpring GX12.5 software. A moderated *t*-test method was applied for assessing the statistically significantly differentially expressed genes. Statistical analysis was performed for the identification of differentially expressed genes between stressed transgene and stressed control experiment groups. Genes showing significant change in the expression level, fold change ≥2 with *P-*value ≤0.05, were identified as differentially expressed genes. The identification of statistically significant enrichment of functional categories in differentially expressed genes was accomplished using DAVID Bioinformatics Resources 6.7.

### Gene expression analysis using quantitative real-time PCR (qRT-PCR)

First-strand cDNA was synthesized from RNA samples of WT and transgenic (RD2) seedlings subjected to mannitol (200mM) stress for 3 d. RT-PCR analysis was carried out using SYBR green master mix with the Applied Biosystems 7500 real-time PCR system at 94 °C (1min), 60 °C (1min), and 72 ° C (1min) for 30 cycles. Later, the products were analysed through a melt curve analysis to check the specificity of PCR amplification. Each reaction was performed three times, and the relative expression ratio was calculated using the 2^–ΔΔct^ method employing the actin gene as the reference. The primers used for qRT-PCR are listed in Supplementary Table S1 available at *JXB* online.

### Statistical analysis

All the experiments were carried out in triplicate employing 20 seedlings in each treatment. Mean values, standard error, and *t*-test were computed with the help of pre-loaded software in Excel, programmed for statistical calculations.

## Results

### Characterization of the *Cajanus cajan* cold and drought regulatory gene (*CcCDR*)

**Fig. 1.**
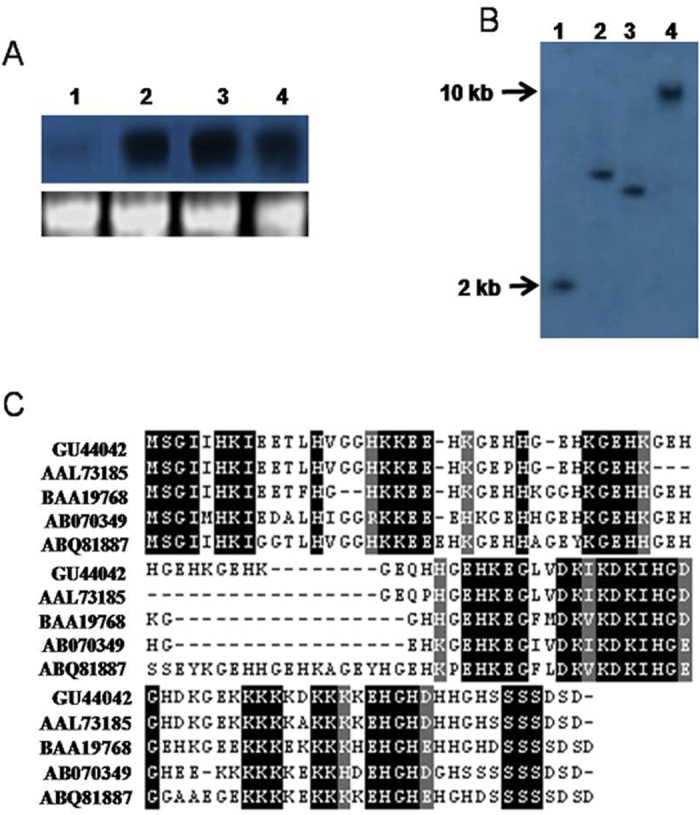
Molecular characterization of the *Cajanus cajan* cold and drought regulatory gene (*CcCDR*) and comparison of its protein product with other plant proteins. (A) Northern blot analysis of *CcCDR*: 4-week-old plants of pigeonpea were subjected to no stress (1); PEG (20%) stress for 6h (2); NaCl (1M) stress for 6h (3); or cold (4 °C) stress for 6h (4). About 20 μg of total RNA was used for northern blot analysis. The blot was hybridized with the cDNA fragment of *CcCDR*. Ethidium bromide-stained 28S rRNA is shown for equal RNA loading. (B) Southern blot analysis of *CcCDR*: lane 1, *Eco*RI-digested; lane 2, *Bam*HI-digested; lane 3, *Hin*dIII-digested; lane 4, *Sal*I-digested genomic DNA of pigeonpea. Positions of 2.0kb and 10.0kb fragments in the gel are indicated. (C) Comparison of the deduced amino acid sequence of CcCDR with other proteins. Multiple sequence alignment of CcCDR (GU444042) with *Carica papaya* maturation-associated like Src1 protein (AAL73185), *Glycine max* cold-induced protein Src1 (BAA19768), *G. max* low temperature-inducible protein (ABO70349), and *G. max* KS-type dehydrin (ABQ81887). Identical and conserved amino acids are represented in black and grey, respectively. (This figure is available in colour at *JXB* online.)

Employing PCR-based cDNA subtraction, a partial cDNA clone (accession no. GD173778) of 200bp was obtained from the cDNA library of pigeonpea plants subjected to 20% PEG stress (–1.01±0.02Mpa). Using 5′ RACE PCR, a full-length cDNA clone (GU444042) was recovered. The clone contained a 282bp coding sequence that codes for a polypeptide of 93 amino acids and was designated as *Cajanus cajan* cold and drought regulatory gene (*CcCDR*). Northern blot analysis of RNA isolated from pigeonpea plants, treated with PEG (20%), NaCl (1M), and cold (4 °C) for 6h, showed increased levels of *CcCDR* transcripts in comparison with the untreated controls (Fig. 1A). Southern blot analysis of genomic DNA, when digested with *Bam*HI, *Eco*RI, *Hin*dIII, and *Sal*I enzymes and probed with the *CcCDR* coding sequence, revealed single hybridization signals of various sizes ranging from >2kb to 10kb (Fig. 1B). The CcCDR protein showed a high identity of 73% with maturation-associated like Src1 protein of *Carica papaya* (AAL73185), 70% with *Glycine max* cold-induced protein Src1 (BAA19768), 68% with *G. max* low temperature-inducible protein (ABO70349), and 59% with *G. max* KS-type dehydrin (ABQ81887) (Fig. 1C).

### Development of *CcCDR-*transgenic plants of *A. thaliana*

Putative transgenic plants obtained with plasmid constructs containing *CcCDR* (Supplementary Fig. S1A at *JXB* online) were selected on MS medium containing kanamycin (50mg l^–1^). PCR analysis using *CcCDR* gene-specific primers revealed amplification of an ~280bp fragment from the genomic DNA of transgenic plants, while no such band was observed in WT and VT plants (data not shown). Southern blot analysis of genomic DNA of transgenic lines demonstrated the presence of the transgene in the genome of *Arabidopsis*, when probed with *CcCDR* (Supplementary Fig. S1B). RT-PCR analysis revealed the presence of *CcCDR* transcripts in rd29A-*CcCDR* lines (subjected to mannitol) and unstressed CaMV35S-*CcCDR* lines (Supplementary Fig. S1C). Homozygous transgenic lines with a single T-DNA insertion, namely CS1 and CS2 (CaMV35S-*CcCDR*) and RD1 and RD2 (rd29A-*CcCDR*), were chosen for subsequent experiments.

### Overexpression of CcCDR in *Arabidopsis* results in enhanced drought, salt, and cold tolerance

**Fig. 2.**
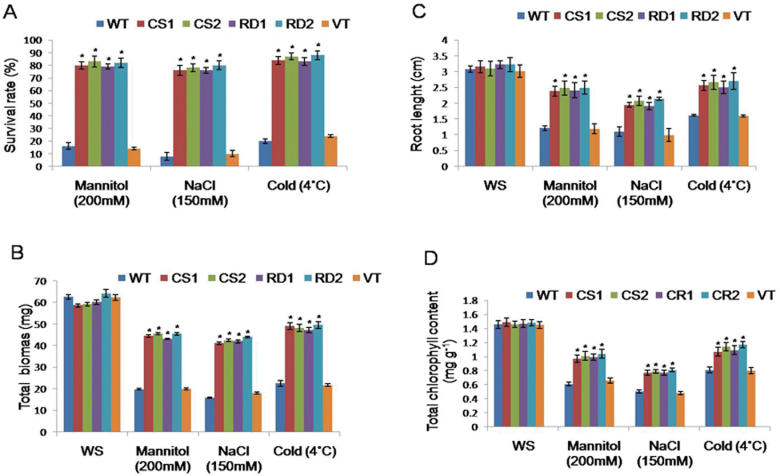
Evaluation of *CcCDR*-transgenics under different abiotic stresses. Two-week-old seedlings of WT, VT, and transgenics were grown under 200mM mannitol, 150mM NaCl, and cold stress (4 °C) for 7 d. Seedlings were allowed to recover on MS plates. Data on survival rate (A), total biomass (B), and root length (C) were recorded after 10 d of recovery. In each treatment, 20 seedlings of WT, VT, and two transgenic lines were used. Chlorophyll content (D) was determined from the leaf discs of control and transgenic plants after 72h of incubation in water, 200mM mannitol, and 150mM NaCl solutions independently at room temperature (20±1 °C); for cold stress, leaf discs were incubated in water at 4 °C. The mean and SE from three independent experiments are shown. * indicates significant differences in comparison with the WT at *P*<0.05. WS, without stress; CS1 and CS2, 35S transgenic lines; RD1 and RD2, rd29A transgenic lines; WT, wild type; VT, vector-transformed plants; FW, fresh weight. (This figure is available in colour at *JXB* online.)

Under different stress conditions, transgenic lines exhibited a higher survival rate as compared with VT and WT (Fig. 2A). Transgenic lines subjected to drought stress showed higher biomass (43.1±0.2mg to 45.5±0.6mg) when compared with VT and WT plants (Fig. 2B). Similarly, transgenic lines subjected to salt stress showed a total biomass of 41.04±0.69mg and 42.4±0.59mg, and 41.98±0.7mg and 43.92±0.3mg compared with VT (15.84±0.2mg) and WT (18.0±0.4mg) plants (Fig. 2B). In comparison with WT and VT plants, *CcCDR*-transgenics exhibited greater root length (2.38±0.1cm to 2.49±0.21cm) under mannitol and NaCl stresses (1.91±0.11cm to 2.14±0.05cm) (Fig. 2C). Likewise, transgenic lines subjected to 4 °C exhibited increased (47.2±1.27mg to 49.5±1.59mg) biomass when compared with VT and WT plants (Fig. 2B). Moreover, the transgenic lines showed increased root length when compared with WT and VT plants (Fig. 2C).

**Fig. 3.**
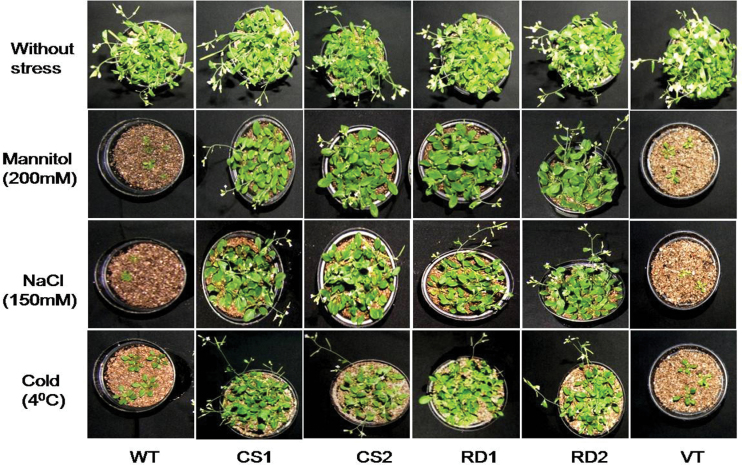
Evaluation of transgenic plants expressing *Cajanus cajan* cold and drought regulatory protein (CcCDR) under different abiotic stress conditions. Two-week-old seedlings of control and *CcCDR*-transgenics were subjected to 200mM mannitol, 150mM NaCl, and cold (4 °C) for 7 d. For each treatment, 20 seedlings were used. Treated seedlings were allowed to recover for 7 d at 20±1 °C. Later, seedlings were transferred to soil and allowed to grow for 3 weeks under normal conditions, and were photographed. (This figure is available in colour at *JXB* online.)

Leaf discs of transgenic lines together with VT and WT plants were floated independently for 72h on water, 200mM mannitol, and 150mM NaCl, solutions at 20±1 °C and also on water at 4 °C for estimation of the total chlorophyll content. Under normal conditions, there were no significant differences in the chlorophyll contents between controls and transgenic plants. However, under stress conditions, the chlorophyll content of WT and VT plants was significantly lower than that of transgenic plants. The mean chlorophyll contents of transgenic plants were found to be higher under mannitol, NaCl, and cold stresses, as compared with WT and VT plants (Fig. 2D). Furthermore, the transgenic plants expressing CcCDR could successfully complete their reproductive cycle, while the control plants turned chlorotic and failed to reach the reproductive phase under drought, salt, and cold stress conditions (Fig. 3).

### Estimation of proline and reducing sugars in *CcCDR*-transgenic plants

**Fig. 4.**
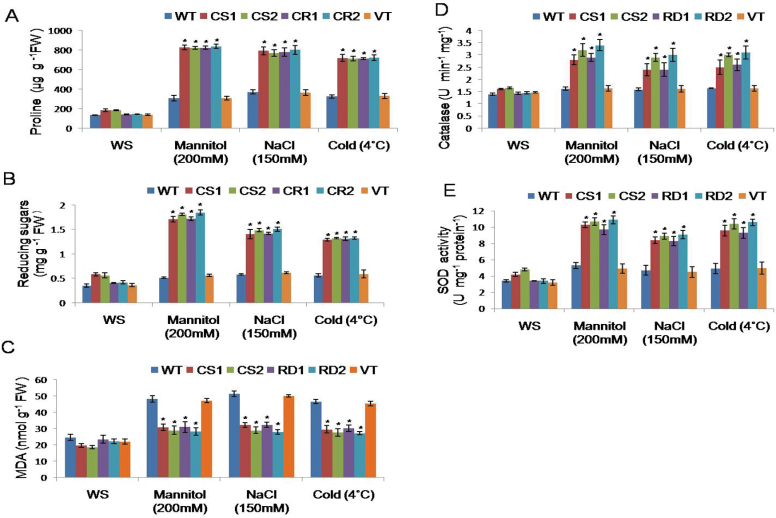
Biochemical characterization of transgenic plants expressing CcCDR. Two-week-old seedlings of control and transgenic plants were grown under 200mM mannitol, 150mM NaCl, and cold stress (4 °C) for 3 d, for estimation of proline (A), reducing sugars (B), MDA (C), catalase (D), and SOD (E). The mean and SE from three independent experiments are shown. For each treatment, 20 seedlings were used. * indicates significant differences in comparison with the WT at *P*<0.05. WT, wild type; VT, vector transformed; CS1 and CS2, 35S transgenic lines; RD1 and RD2, rd29A transgenic lines. (This figure is available in colour at *JXB* online.)

Under normal conditions, CS1 and CS2 transgenic lines showed higher proline and reducing sugar contents. However, there was no significant difference between proline and reducing sugars of RD transgenic, WT, and VT plants (Fig. 4A, B). When 15-day-old transgenics were subjected to mannitol (200mM) stress, transgenic plants accumulated higher levels of proline and reducing sugars, respectively (Fig. 4A, B). In contrast, WT and VT plants showed lower levels of proline and reducing sugars, under similar stress conditions. After NaCl (150mM) stress, transgenic plants accumulated higher levels of proline and reducing sugars, while VT and WT plants showed lower levels. Likewise, under cold (4 °C) stress, both the transgenic lines exhibited higher levels of proline and reducing sugars. Under similar conditions, WT and VT plants exhibited lower proline and reducing sugar contents (Fig. 4A, B).

### MDA content in transgenic plants

The MDA content of transgenic plants subjected to mannitol and NaCl stress conditions was significantly lower than that of WT and VT plants. Cold stress enhanced the levels of MDA in WT and VT plants as compared with the transgenic plants (Fig. 4C).

### Catalase activity in transgenic plants

**Fig. 5.**
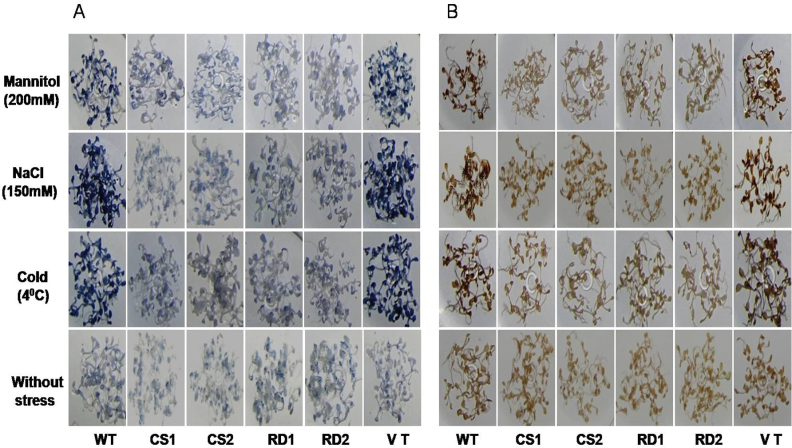
Hydrogen peroxide and superoxide detection in transgenic plants subjected to different abiotic stresses. Two-week-old *A. thaliana* seedlings grown on MS medium were subjected to mannitol (200mM), NaCl (150mM, and cold (4 °C) for 72h. For each treatment, 20 seedlings were used. (A) NBT staining for superoxide detection (B) DAB staining for H_2_O_2_ detection. WT, wild type; VT, vector control; CS1 and CS2, 35S transgenic lines; RD1 and RD2, rd29A transgenic lines. (This figure is available in colour at *JXB* online.)

Catalase activity in transgenic seedlings under mannitol-, NaCl-, and cold-induced stress conditions was higher as compared with WT and VT plants (Fig. 4D). Further, under such stress conditions, the intensity of DAB staining in control plants was stronger than that of transgenic seedlings (Fig. 5A).

### SOD activity in transgenic plants

SOD activity was assayed in the WT, VT, and transgenic seedlings in the presence and absence of stress conditions. The SOD activity was significantly increased in the transgenics upon exposure to mannitol, NaCl, and cold stress conditions compared with activities recorded in WT and VT plants (Fig. 4E). Furthermore, the relative superoxide concentration was examined with NBT staining. Under normal conditions, transgenic and control seedlings showed low staining, while transgenics exhibited a lower intensity of staining than control seedlings under stress conditions (Fig. 5B).

### Cell membrane stability of transgenic *Arabidopsis* plants

**Fig. 6.**
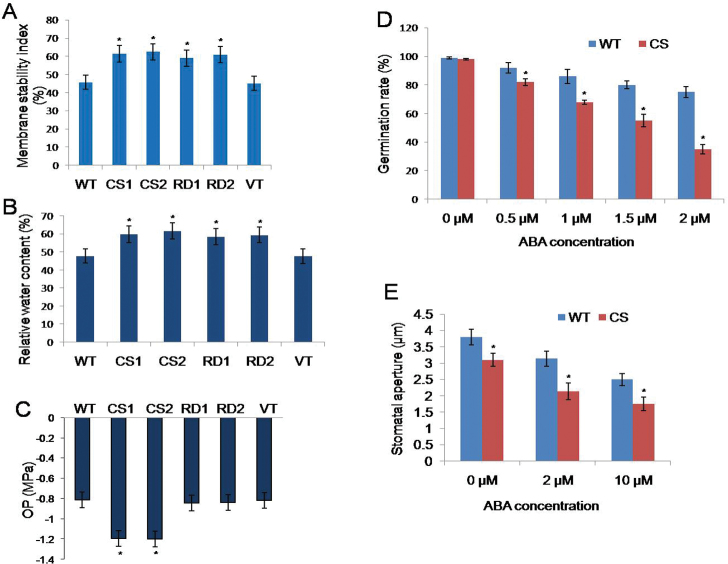
Physiological characterization of *CcCDR*-transgenic plants. (A) Cell membrane stability measurement of transgenics, WT, and VT seedlings treated with 150 mM NaCl. (B) Comparison of relative water contents of detached rosettes of controls and transgenic plants. (C) Measurement of osmotic potential of transgenic lines, WT, and VT plants under well-watered conditions. (D) Germination rates of transgenic (CS) and WT seeds on ABA-containing medium. Seeds were placed on MS medium containing 0, 0.5, 1.0, 1.5, or 2.0 μM ABA and the germination rate was calculated after 7 d. Seeds were considered to have germinated when the radicle tip had expanded by 1mm. (E) Size of stomatal apertures of transgenics (CS) and the WT after ABA treatment. Leaves of transgenics and the WT were treated with ABA- (2.0 μM and 10.0 μM) containing solution for 2h. Stomatal apertures in epidermal peels were observed under a confocal microscope and 50 stomatal apertures were measured for each treatment. The mean and SE from three independent experiments are shown. For each treatment, 20 seedlings were used. * indicates significant differences in comparison with the WT at *P*<0.05. WT, wild type; VT, vector transformed; CS1 and CS2, 35S transgenic lines; RD1 and RD2, rd29A transgenic lines. (This figure is available in colour at *JXB* online.)

Eight-day old seedlings of transgenic, WT, and VT plants were treated with NaCl (150mM) for analysis of their cell membrane stability. After 6h of stress, control plants exhibited wilting symptoms, while transgenics showed no signs of stress-induced symptoms. The results revealed that the cell membrane stability of CS1 and CS2 (34% and 36%) and RD1 and RD2 (29% and 33%) transgenics was significantly higher than that of WT and VT plants (Fig. 6A). Moreover, no significant differences were observed between CS and RD transgenic lines under similar stress conditions.

### Water retention capacity of transgenic plants

To assess the water retention ability of transgenics, the fresh water loss in detached rosette leaves was measured. Although water losses were observed in transgenic and control plants, the final relative water content of CS1 and CS2 (25% and 28%) and RD1 and RD2 (22% and 24%) transgenics was significantly higher than that of WT and VT plants (Fig. 6B).

### Analysis of osmotic potential of *CcCDR*-transgenics

To determine the role of CcCDR in stress tolerance, the osmotic potential of transgenic, VT, and WT plants grown under well-watered conditions was measured. The osmotic potential of CS1 and CS2 lines was higher (46% and 47%) than that of RD1 and RD2, VT, and WT plants (Fig. 6C). Furthermore, no significant differences were observed in the osmotic potential of RD, VT, and WT plants.

### ABA sensitivity of *CcCDR*-transgenic plants

The seed germination rate of transgenic plants was severely inhibited by ABA in comparison with the WT plants (Fig. 6D). Moreover, transgenic seedlings showed higher hypersensitivity to ABA (Supplementary Fig. S2 at *JXB* online). The density of guard cells was comparable in both transgenics and WT pants. However, when treated with ABA, transgenic plants showed a greater reduction in stomatal aperture size compared with WT plants (Fig. 6E).

### Localization of CcCDR protein in stably transformed cells

**Fig. 7.**
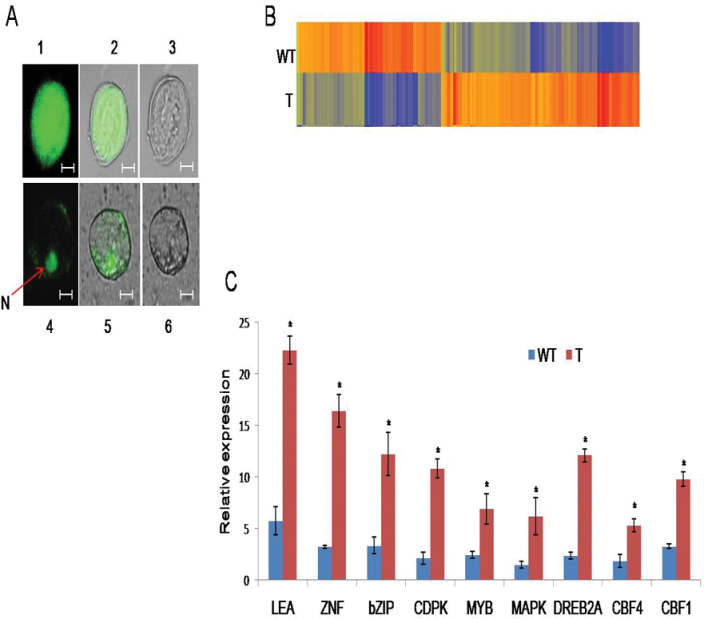
Subcellular localization and expression profile of CcCDR in transgenic *Arabidopsis.* (A) Subcellular localization of CcCDR. Plasmid constructs harbouring CaMV35S-*GFP* and CaMV35S-*CcCDR::GFP* were introduced into *Arabidopsis* independently by floral infiltration. Individual cells of callus derived from roots of transgenic plants were observed under a confocal microscope. Images 1 and 4 are dark field, 2 and 5 are combined, 3 and 6 are bright field. Ths scale bar=50 μm. N represents the nucleus. (B) Transcriptome profile of wild-type and rd29A-*CcCDR*-transgenic plants under drought stress by microarray analysis. Two-week-old seedlings were subjected to 200mM mannitol stress for 72h. The heat map represents normalized expression intensity values of differentially regulated genes (fold change ≥2 and *P*-value <0.05) in the RD2 transgenic line (T) as compared with the wild type (WT). Dark color shows overexpressed genes and light color shows underexpressed genes. (C) Expression profiles of stress-responsive genes under drought stress by qRT-PCR analysis. Comparisons of the relative transcript levels of LEA, ZNF, bZIP, CDPK, MYB, MAPK, DREB2A, CBF4, and CBF1 in rd29A-*CcCDR-*transgenic (T) and wild-type (WT) plants and under 200mM mannitol stress for 72h. Actin was used as an internal control. The vertical column indicates the relative transcript level. The mean and SE from three independent experiments are shown. * indicates significant differences in comparison with the control at *P*<0.05. (This figure is available in colour at *JXB* online.)

To examine the subcellular localization of CcCDR protein in the cells, the *CcCDR*::*GFP* fusion construct and *GFP* (control) driven by the CaMV 35S promoter were stably expressed in *Arabidopsis*. Cells containing CcCDR::GFP showed substantial fluorescence in the nucleus as well as its presence in the cytoplasm, while cells expressing GFP alone exhibited uniform fluorescence throughout the cell (Fig. 7A).

### Microarray analysis of rd29A-*CcCDR* plants under drought stress conditions

To elucidate the consequence of CcCDR overexpression, microarray analysis of WT and RD transgenic (rd29A-*CcCDR*) plants subjected to mannitol (200mM) stress was performed. The fold change analysis (FC ≥2.0) showed that 3267 genes were expressed >2-fold in the transgenic as compared with WT plants, of which 1780 were 2-fold up-regulated and 1487 were 2-fold down-regulated. Furthermore, the fold-change analysis (FC ≥4.0) revealed that 409 genes were up-regulated >4-fold (Supplementary Table S2 at *JXB* online) and 297 genes were down-regulated <4-fold (data not shown). In *CcCDR*-transgenics, the number of up-regulated genes was higher compared with down-regulated genes. The heat map image of Hierarchical CLustering (HCL) showed overexpressed genes in a dark colour and underexpressed genes in a light colour (Fig. 7B).

### Expression analysis of stress-responsive genes in rd29a-*CcCDR* transgenic plants by qRT-PCR

To confirm the results obtained from the microarray analysis, nine genes which showed >4-fold change were selected for qRT-PCR analysis. The results revealed substantial increases in the relative expression of these genes, namely LEA, ZNF, bZIP, CDPK, MYB, MAPK, DREB2A, and CBF4, in transgenic *Arabidopsis* plants under drought stress conditions. However, the expression levels of these genes were significantly lower in the WT plants under similar conditions (Fig. 7C).

## Discussion

In this study, a stress-responsive *CcCDR* gene was isolated from the subtractive cDNA library of pigeonpea plants subjected to drought stress. Southern blot analysis showed the presence of single hybridizable bands of various sizes, denoting the single-copy nature of *CcCDR* and the absence of paralogues in the pigeonpea genome. Northern blot analysis, using the RNA isolated from plants exposed to PEG, NaCl, and cold treatments, revealed intense *CcCDR* signals when compared with untreated pigeonpea plants, suggesting the stress-responsive nature of the gene. Although CcCDR did not show complete homology with any of the proteins associated with abiotic stress tolerance, it still showed ~70% homology with the cold-induced Src1 protein of *G. max* (Fig. 1C). In soybean, the accumulation of *Src*1 transcripts was significantly increased in a tissue-specific manner on exposure to low temperature and drought conditions (Takahashi and Shimosaka, 1997). These results suggest that *CcCDR* might have originated and diverged from a common source during the course of evolution to cope with different environmental conditions.

Under different stress conditions, *CcCDR-*transgenics exhibited a higher survival rate, increased root length, enhanced plant biomass, and higher chlorophyll content when compared with WT and VT plants (Fig. 2), as well as normal fertility. The striking differences observed between transgenic and control plants demonstrate the profound effect of CcCDR in bestowing multiple abiotic stress tolerance at the whole-plant level (Fig. 3). Under stress conditions, transgenic plants exhibited higher RWCs when compared with WT and VT plants, thereby indicating that transgenics have higher water retention ability. Water loss rate is an important parameter which reflects the water status of the plant and has been used as a reliable indicator of drought tolerance in plants (Dhanda and Sethi, 1998; Song *et al.*, 2012). Transgenic *Arabidopsis* expressing vacuolar H^+^ pyrophosphatase (AVP1H^+^) and wheat protein kinase TaABC1 exhibited increased ability for water retention as compared with WT plants (Gaxiola *et al.*, 2001; Wang *et al.*, 2010). *CcCDR*-transgenic plants also showed higher cell membrane stability values when compared with WT and VT plants. Cell membranes are thought to be the primary target, and their stability and integrity are of primary concern in plants grown under abiotic stress conditions (Wang *et al.*, 2010). Wheat genes, TaSnRK2.4 and TaABC1, when overexpressed in *Arabidopsis*, caused increased cell membrane stability under stress conditions (Mao *et al.*, 2009; Wang *et al.*, 2010). Under unstressed conditions, the osmotic potential of transgenic plants expressing CcCDR driven by the CaM V35 promoter was found to be higher than that of WT and VT plants, indicating the unequivocal role of CcCDR in osmotic potential adjustment. Overexpression of wheat Na^+^/H^+^ antiporter TNHX1 and H^+^-pyrophosphatase TVP1 in *Arabidopsis* resulted in an enhanced osmotic adjustment compared with control plants (Brini *et al.*, 2007).

An increase in the free proline content was evident in *CcCDR-*transgenic lines when compared with WT and VT plants. In general, the osmotic potential increases due to the accumulation of osmoprotectants such as sugars and amino acids, and thus plants accumulate free proline under stress conditions. Proline not only acts as an osmoprotectant but also serves as a free radical scavenger and protects macromolecules against denaturation in the cell. Furthermore, overproduction of proline in plants increases their osmotic stress tolerance (Kishor *et al.*, 1995). Generally, plants with higher capacity for osmotic adjustment showed broader tolerance to osmotic stress (Mao *et al.*, 2009). *CcCDR*-transgenic plants also exhibited enhanced accumulation of reducing sugars when compared with WT and VT plants. Accumulation of reducing sugars is advantageous due to their high water solubility and resistance to crystallization at freezing temperatures. They also stabilize membranes and indirectly contribute to the osmotic adjustment upon freezing and dehydration (Krasensky and Jonak, 2012). In rice, overexpression of *Os*CIPK12 caused accumulation of higher proline and soluble sugars as compared with control plants (Xiang *et al.*, 2007).

In the present study, lower levels of MDA were observed in transgenic lines under drought, salt, and cold treatments when compared with WT and VT plants (Fig. 4C). These results were further substantiated by the presence of low levels of superoxide in the transgenic plants compared with WT and VT plants as evidenced by the NBT staining (Fig. 5B), suggesting lower levels of lipid peroxidation in transgenic plants. Reactive oxygen species (ROS) produced in plants under drought, salt, and temperature stress conditions cause oxidative stress (Mittler *et al.*, 2004), which leads to an accumulation of higher levels of MDA due to increased concentration of superoxide radicals, resulting in damage to cell membranes. Tobacco plants overexpressing finger millet NAC1 (EcNAC1) transcription factor showed lower lipid peroxidation due to the lower levels of MDA under abiotic stress conditions (Ramegowda *et al.*, 2012).

Analysis of catalase and SOD under stress conditions revealed higher levels of activity in trasnsgenics as compared with WT and VT plants. Furthermore, *CcCDR*-transgenic plants showed lower levels of H_2_O_2_ and superoxide when compared with the control plants, as evidenced by DAB and NBT staining (Fig. 5). Different ROS, such as ^1^O_2_, H_2_O_2_, O_2_^–^, and HO·, are toxic molecules and cause oxidative damage to vital molecules of the cell (Apel and Hart, 2004). During stress conditions, the rate of ROS production increases significantly and ROS-scavenging enzymes, namely catalase, SOD, ascorbate peroxidase, glutathione peroxidase, and peroxiredoxin, are essential for their detoxification (Mittler *et al.*, 2004). Catalase is an important antioxidant enzyme which catalyses the decomposition of H_2_O_2_ and plays a major role in controlling homeostasis of ROS (Du *et al.*, 2008). SOD is the first enzyme acting in detoxification and converts O_2_· radicals to H_2_O_2_. It was reported that overexpression of Fe-SOD in alfalfa improved the winter survival of transgenics due to its increasing superoxide-scavenging capacity (McKersie *et al.*, 2000). Overexpression of the *Arabidopsis* MAPK kinase gene *AtMEK1* significantly increased stress-induced CAT1 expression which reduced the superoxide concentration in transgenic plants (Xing *et al.*, 2007). The results of NBT and DAB tests indicate the efficient scavenging of ROS in CcCDR-expressing transgenic plants.

*Arabidopsis* transgenics expressing CcCDR were found to be hypersensitive to ABA at both seed and vegetative stages. These results clearly indicate that CcCDR functions as a transcriptional activator of ABA signal transduction, which is responsible for tolerance to multiple abiotic stresses. *Arabidopsis* plants overexpressing AtMYC2 and AtMYB2 proteins exhibited hypersensitivity to ABA due to transcriptional activation of ABA-inducible gene expression (Abe *et al.*, 2003). Further, CcCDR-transgenic plants revealed lower stomatal aperture size as compared with WT plants when treated with ABA (Fig. 6E). Stomatal closure is a key ABA-controlled process for coping during water deficit conditions. As such, CcCDR might play a role in the ABA-induced stomatal closure which results in enhanced stress tolerance. Transgenic *Arabidospsis* overexpressing AtMYB44 revealed enhanced stomatal closure and conferred abiotic stress tolerance (Jung *et al.*, 2008).

To determine the nature of accumulation of CcCDR protein in the cell, the *CcCDR–GFP* fusion construct was stably expressed in transgenic *Arabidopsis*. When cells expressing CcCDR–GFP were analysed, the fusion protein was found to be localized predominantly in the nucleus (Fig. 7A), indicating that CcCDR enters into the nucleus and might interact with other nuclear proteins to regulate the expression of various stress-responsive genes.

Microarray analysis was carried out to obtain evidence for the role of CcCDR in the regulation of abiotic stress-responsive genes as well as to understand the multiple stress protection capabilities of *CcCDR* transgenic plants. The results obtained indicate that many genes involved in abiotic stress response showed higher expression levels in rd29A-*CcCDR*-transgenic plants as compared with WT plants under similar stress conditions (Supplementary Table S2 at *JXB* online). Genes that have physiological and molecular functions under abiotic stresses, such as DREB2A, zinc finger (C2H2 type) protein, MYB, MAPKKK, CPKs, CBF1, CBF4, bZIP transcription factor, and LEA, were up-regulated in *CcCDR*-transgenic plants. qRT-PCR analysis also confirmed the up-regulation of these stress-responsive genes in transgenic plants expressing CcCDR under similar stress conditions (Fig. 7C). MYB and bZIP family proteins function as transcriptional activators in the ABA signal transduction pathway under drought stress conditions (Abe *et al.*, 2003). Up-regulation of the *A. thaliana* 9-*cis*-epoxycarotenoid dioxygenase (*NCED*2) gene, which codes for a key enzyme in ABA biosynthesis, was also detected in *CcCDR*-transgenic plants under drought stress conditions. It is well known that ABA mediates a variety of physiological processes including the response to drought, salt, and cold stress (Zhu *et al.*, 2007). Most of the drought-inducible genes studied to date are also induced by ABA. Up-regulation of transcripts of these genes in transgenic plants indicates the role of CcCDR in activating the ABA-dependent pathway. Under stress conditions, the expression levels of LEA and P5CS were up-regulated, which is consistent with the higher proline content and osmotic potential of the transgenic plants. Similarly, increased expression of the C2H2-zinc finger protein in the transgenic plants indicates its possible role in enhanced stress tolerance. C2H2-zinc finger proteins participate in various abiotic stresses, such as salt, osmotic, drought, and low temperature stress, by enhancing the expression of different ROS response transcripts, thereby providing tolerance capabilities (Mittler *et al.*, 2006). Overexpression of a rice zinc-finger protein gene (*OSISAP1*) in tobacco conferred tolerance to cold, dehydration, and salt stress (Mukhopadhyay *et al.*, 2004).

Up-regulation of CDPKs and MAPKs as well as increases in antioxidant enzyme levels in transgenic plants indicate that the *CcCDR* gene plays an important role in ABA-mediated stress tolerance pathways. CDPKs act upstream of MAPK and are involved in enhancing the expression levels of antioxidants such as catalase, ascorbate peroxidase, glutathione reductase, and SOD (Ding *et al*., 2012). Furthermore, CDPKs and MAPKs are positive regulators of ABA signalling processes, regulating many aspects of plant growth and development as well as plant adaptation to biotic and abiotic stresses (Zhu *et al.*, 2007; Asano *et al.*, 2012).

A significant increase in DREB2A transcripts in the transgenic plants might have resulted in increased expression levels of downstream drought stress- and cold-responsive genes owing to cross-talk between signalling networks operating under different stress conditions. DREB2A overexpression regulates many water stress-inducible genes, leading to significant drought stress tolerance in *Arabidopsis* under stress conditions (Sakuma *et al.*, 2006). Moreover, transcript levels of CBF1 and CBF4 were significantly higher in *CcCDR*-transgenic plants than in WT plants. The CBF1 gene is mainly involved in ABA-independent regulation of stress-responsive genes (Nakashima *et al.*, 2009), while the CBF4 gene (a homologue of CBF/DREB1) is rapidly induced during drought stress and by ABA treatment, and regulates stress-responsive gene expression in an ABA-dependent manner (Haake *et al.*, 2002). These results suggest that both ABA-dependent and ABA-independent stress signalling pathways are involved in CcCDR-mediated stress tolerance. Hence, it may be presumed that CcCDR acts upstream of these stress-responsive genes and is, therefore, involved in a cross-talk between both the signalling networks. Based on the overall results, it is presumed that the enhanced abiotic stress tolerance of *Arabidopsis* transgenic plants is due to a significant increase in the expression levels of different abiotic stress-responsive genes.

## Supplementary data

Suplementary data are available at *JXB* online.

Figure S1. Molecular analysis of *CcCDR*-transgenic *Arabidopsis* plants.

Figure S2. Twenty-day-old WT and transgenic (T) *Arabidopsis* seedlings grown on MS medium containing 1.5 μM ABA.

Table S1. Selected genes and primers used for real-time PCR analysis.

Table S2. List of genes up-regulated >4-fold in *CcCDR* transgenic *Arabidopsis* subjected to drought stress.

Supplementary Data

## References

[CIT0001] Abe H Urao T Ito T Seki M Shinozaki K Yamaguchi-Shinozaki K 2003 Arabidopsis AtMYC2 (bHLH) and AtMYB2 (MYB) function as transcriptional activators in abscisic acid signaling. The Plant Cell 15, 63–78 12509522 10.1105/tpc.006130 PMC143451

[CIT0002] Apel K Hirt H 2004 Reactive oxygen species: metabolism, oxidative stress, and signal transduction. Annual Review of Plant Biology 55, 373–399 10.1146/annurev.arplant.55.031903.141701 15377225

[CIT0003] Asano T Hayashi N Kobayashi M 2012 A rice calcium-dependent protein kinase OsCPK12 oppositely modulates salt-stress tolerance and blast disease resistance. The Plant Journal 69, 26–36 21883553 10.1111/j.1365-313X.2011.04766.x

[CIT0004] Bates LS Waldran R Teare ID 1973 Rapid determination of free proline for water studies. Plant and Soil 39, 205–208

[CIT0005] Brini F Hanin M Mezghani I Berkowitz GA Masmoudi K 2007 Overexpression of wheat Na+/H+ antiporter TNHX1 and H+-pyrophosphatase TVP1 improve salt- and drought stress tolerance in *Arabidopsis thaliana* plants. Journal of Experimental Botany 58, 301–308 17229760 10.1093/jxb/erl251

[CIT0006] Clough SJ Bent AF 1998 Floral dip: a simplified method for *Agrobacteriu*m-mediated transformation of *Arabidopsis thaliana* . The Plant Journal 16, 735–743 10069079 10.1046/j.1365-313x.1998.00343.x

[CIT0007] Dhanda SS Sethi GS 1998 Inheritance of excised-leaf water loss and relative water content in bread wheat (*Triticum aestivum*). Euphytica 104, 39–47

[CIT0008] Ding Y Cao J Ni L Zhu Y Zhang A. Tan M Jiang M 2013 ZmCPK11 is involved in abscisic acid-induced antioxidant defense and functions upstream of ZmMPK5 in abscisic acid signaling in maize. Journal of Experimental Botany 64, 871–884 23268839 10.1093/jxb/ers366 PMC3580805

[CIT0009] Du Y Wang P Chen J Song CP 2008 Comprehensive functional analysis of the catalase gene family in *Arabidopsis thaliana* . Journal of Integrative Plant Biology 50, 1318–1326 19017119 10.1111/j.1744-7909.2008.00741.x

[CIT0010] Feng DR Liu B Li WY He YM Qi KB Wang BH Wang FJ 2009 Over-expression of a cold-induced plasma membrane protein gene (*MpRCI*) from plantain enhances low temperature-resistance in transgenic tobacco. Environmental and Experimental Botany 65, 395–402

[CIT0011] Fowler S Thomashow MF 2002 Arabidopsis transcriptome profiling indicates that multiple regulatory pathways are activated during cold acclimation in addition to the CBF cold response pathway. The Plant Cell 14, 1675–1690 12172015 10.1105/tpc.003483 PMC151458

[CIT0012] Gaxiola RA Li JS Undurraga S Dang LM Allen GJ Alper SL Fink GR 2001 Drought and salt tolerant plants result from overexpression of the AVP1 H+-pump. Proceedings of the National Academy of Sciences, USA 98, 11444–11449 10.1073/pnas.191389398 PMC58749 11572991

[CIT0013] Gupta A S Webb RP Holaday AS Allen RD 1993 Over-expression of superoxide dismutase protects plants from oxidative stress: induction of ascorbate peroxidase in superoxide dismutase-overexpressing plants. Plant Physiology 103, 1067–1073 12232001 10.1104/pp.103.4.1067 PMC159091

[CIT0014] Haake V Cook D Riechmann JL Pineda O Thomashow MF Zhang JZ 2002 Transcription factor CBF4 is a regulator of drought adaptation in Arabidopsis. Plant Physiology 130, 639–648 12376631 10.1104/pp.006478 PMC166593

[CIT0015] Jung C Seo JS Han SW Koo YJ Kim CH Song SI Nahm BH Choi YD Cheong JJ 2008 Overexpression of AtMYB44 enhances stomatal closure to confer abiotic stress tolerance in transgenic Arabidopsis. Plant Physiology 146, 623–635 18162593 10.1104/pp.107.110981 PMC2245844

[CIT0016] Kasuga M Liu Q Miura S Yamaguchi-Shinozaki K Shinozaki K 1999 Improving plant drought, salt, and freezing tolerance by gene transfer of a single stress-inducible transcription factor. Nature Biotechnology 17, 287–291 10.1038/7036 10096298

[CIT0017] Kishor KPB Hong Z Miao GH Hu CAA Verma DPS 1995 Overexpression of D1-pyrroline-5-carboxylate synthetase increases proline production and confers osmotolerance in transgenic plants. Plant Physiology 108, 1387–1394 12228549 10.1104/pp.108.4.1387 PMC157516

[CIT0018] Krasensky J Jonak C 2012 Drought, salt, and temperature stress-induced metabolic rearrangements and regulatory networks. Journal of Experimental Botany 63, 1593–1608 22291134 10.1093/jxb/err460 PMC4359903

[CIT0019] Mao X Zhang H Tian S Chang X Jing R 2009 TaSnRK2.4, an SNF1-type serine-threonine protein kinase of wheat (*Triticum aestivum* L.) confers enhanced multi-stress tolerance in Arabidopsis. Journal of Experimental Botany 61, 683–696 20022921 10.1093/jxb/erp331 PMC2814103

[CIT0020] McKersie BD Murnaghan J Jones KS Bowley SR 2000 Iron-superoxide dismutase expression in transgenic alfalfa increases winter survival without a detectable increase in photosynthetic oxidative stress tolerance. Plant Physiology 122, 1427–1437 10759538 10.1104/pp.122.4.1427 PMC58977

[CIT0021] Miller GL 1959 Use of dinitrosalicylic acid reagent for determination of reducing sugars. Analytical Chemistry 31, 426–428

[CIT0022] Mittler R Kim Y Song L Coutu J Coutu A Ciftci-Yilmaz S Lee H Stevenson B Zhu JK 2006 Gain- and loss-of-function mutations in Zat10 enhance the tolerance of plants to abiotic stress. FEBS Letters 580, 6537–6542 17112521 10.1016/j.febslet.2006.11.002 PMC1773020

[CIT0023] Mittler R Vanderauwera S Gollery M Van Breusegem F 2004 Reactive oxygen gene network of plants. Trends in Plant Science 9, 490–498 15465684 10.1016/j.tplants.2004.08.009

[CIT0024] Mukhopadhyay A Vij S Tyagi AK 2004 Overexpression of a zinc-finger protein gene from rice confers tolerance to cold, dehydration, and salt stress in transgenic tobacco. Proceedings of the National Academy of Sciences, USA 101, 6309–6314 10.1073/pnas.0401572101 PMC395965 15079051

[CIT0025] Murashige T Skoog F 1962 A revised medium for rapid growth and bioassays with tobacco tissue cultures. Plant Physiology 15, 473–497

[CIT0026] Nakashima K Ito Y Yamaguchi-Shinozaki K 2009 Transcriptional regulatory networks in response to abiotic stresses in Arabidopsis and grasses. Plant Physiology 149, 88–95 19126699 10.1104/pp.108.129791 PMC2613698

[CIT0027] Nieva C Busk PK Dominguez-Puigjaner E Lumbreras V Testillano PS Risueno MC Pages M 2005 Isolation and functional characterisation of two new bZIP maize regulators of the ABA responsive gene rab 28. Plant Molecular Biology 58, 899 –914 16240181 10.1007/s11103-005-8407-x

[CIT0028] Park JA Cho SK Kim JE Chung HS Hong JP Hwang B Hong CB Kim WT 2003 Isolation of cDNAs differentially expressed in response to drought stress and characterization of the Ca-LEAL1 gene encoding a new family of atypical LEA-like protein homologue in hot pepper (*Capsicum annuum* L. cv. Pukang). Plant Science 165, 471–481

[CIT0029] Priyanka B Sekhar K Reddy VD Rao KV 2010 Expression of pigeonpea hybrid-proline-rich protein encoding gene (*CcHyPRP*) in yeast and Arabidopsis affords multiple abiotic stress tolerance. Plant Biotechnology Journal 8, 76–87 20055960 10.1111/j.1467-7652.2009.00467.x

[CIT0030] Ramegowda V Senthil-Kumar M Nataraja KN Reddy MK Mysore KS Udayakumar M 2012 Expression of a finger millet transcription factor, EcNAC1, in tobacco confers abiotic stress-tolerance. PLoS One 7, e40397 22808152 10.1371/journal.pone.0040397 PMC3394802

[CIT0031] Saghai-Maroof MA Soliman KM Jorgensen RA Allard RW 1984 Ribosomal DNA spacer-length polymorphisms in barley: Mendelian inheritance, chromosomal location and population dynamics. Proceedings of the National Academy of Sciences, USA 81, 8014–8019 10.1073/pnas.81.24.8014 PMC392284 6096873

[CIT0032] Sakuma Y Maruyama K Osakabe Y Qin F Seki M Shinozaki K Yamaguchi-Shinozaki K 2006 Functional analysis of an Arabidopsis transcription factor DREB2A, involved in drought responsive gene expression. The Plant Cell 18, 1292–1309 16617101 10.1105/tpc.105.035881 PMC1456870

[CIT0033] Sambrook J Russell DW 2001 *Molecular cloning a laboratory manual*. Cold Spring Harbor, NY: Cold Spring Harbor Laboratory Press

[CIT0034] Sekhar K Priyanka B Reddy VD Rao KV 2010 Isolation and characterization of a pigeonpea cyclophilin (*CcCYP*) gene, and its over-expression in *Arabidopsis* confers multiple abiotic stress tolerance. Plant, Cell and Environment 33, 1324–1338 10.1111/j.1365-3040.2010.02151.x 20374537

[CIT0035] Shin LJ Lo JC Yeh KC 2012 Copper chaperone antioxidant protein1 is essential for copper homeostasis. Plant Physiology 159, 1099–1110 22555879 10.1104/pp.112.195974 PMC3387697

[CIT0036] Shinozaki K Yamaguchi-Shinozaki K 1997 Gene expression and signal transduction in water-stress response. Plant Physiology 115, 327–334 12223810 10.1104/pp.115.2.327 PMC158490

[CIT0037] Song S Chen Y Zhao M Zhang WH 2012 A novel *Medicago truncatula* HD-Zip gene, MtHB2, is involved in abiotic stress responses. Environmental and Experimental Botany 80, 1–9

[CIT0038] Takahashi R Shimosaka E 1997 cDNA sequence analysis and expression of two cold-regulated genes in soybean. Plant Science 123, 93–104

[CIT0039] Takasaki H Maruyama K Kidokoro S Ito Y Fujita Y Shinozaki K Yamaguchi-Shinozaki K Nakashima K 2010 The abiotic stress-responsive NAC-type transcription factor OsNAC5 regulates stress-inducible genes and stress tolerance in rice. Molecular Genetics and Genomics 284, 173–183 20632034 10.1007/s00438-010-0557-0

[CIT0040] Tong S Ni Z Peng H Dong G Sun Q 2007 Ectopic overexpression of wheat TaSrg6 gene confers water stress tolerance in Arabidopsis. Plant Science 172, 1079–1086

[CIT0041] Umezawa T Fujita M Fujita Y Yamaguchi Shinozaki K Shinozaki K 2006 Engineering drought tolerance in plants: discovering and tailoring genes to unlock the future. Current Opinion in Biotechnology 17, 113–122 16495045 10.1016/j.copbio.2006.02.002

[CIT0042] Wang C Jing R Mao X Chang X Li A 2010 TaABC1, a member of the activity of bc1 complex protein kinase family from common wheat, confers enhanced tolerance to abiotic stresses in Arabidopsis. Journal of Experimental Botany 63, 1299–1311 21115661 10.1093/jxb/erq377 PMC3022413

[CIT0043] Xiang Y Huang YM Xiong LZ 2007 Characterization of stress responsive CIPK genes in rice for stress tolerance improvement. Plant Physiology 144, 1416–1428 17535819 10.1104/pp.107.101295 PMC1914128

[CIT0044] Xiao B Huang Y Tang N Xiong L 2007 Over-expression of a LEA gene in rice improves drought resistance under the field conditions. Theoretical and Applied Genetics 115, 35–46 17426956 10.1007/s00122-007-0538-9

[CIT0045] Xing Y Jia W Zhang J 2007 AtMEK1 mediates stress-induced gene expression of CAT1 catalase by triggering H_2_O_2_ production in Arabidopsis. Journal of Experimental Botany 58, 2969–2981 17728292 10.1093/jxb/erm144

[CIT0046] Xiong L Schumaker KS Zhu JK 2002 Cell signaling during cold, drought, and salt stress. The Plant Cell 14 (Suppl), S165–S183 12045276 10.1105/tpc.000596 PMC151254

[CIT0047] Xiong L Zhu JK 2003 Regulation of abscisic acid biosynthesis. Plant Physiology 133, 29–36 12970472 10.1104/pp.103.025395 PMC523868

[CIT0048] Zhang H Mao X Wang C Jing R 2010 Overexpression of a common wheat gene TaSnRK2.8 enhances tolerance to drought, salt and low temperature in Arabidopsis. PLoS One 5, e16041 21209856 10.1371/journal.pone.0016041 PMC3012728

[CIT0049] Zhu SY Yu XC Wang XJ Zhao R Li Y Fan C R Shang Y Du SY Wang XF Wu FQ 2007 Two calcium-dependent protein kinases, CPK4 and CPK11, regulate abscisic acid signal transduction in Arabidopsis. The Plant Cell 19, 3019–3036 17921317 10.1105/tpc.107.050666 PMC2174700

