# Supplementary material for: Overexpression of pigeonpea stress-induced cold and drought regulatory gene (CcCDR) confers drought, salt, and cold tolerance in Arabidopsis
Source: J Exp Bot. 2014 May 27;65(17):4769–81. doi: 10.1093/jxb/eru224 (PMC4144763; doi:10.1093/jxb/eru224)
Supplement: Supplementary Data [file supp_65_17_4769__index.html]

Overexpression of pigeonpea stress-induced cold and drought regulatory gene (CcCDR) confers drought, salt, and cold tolerance in Arabidopsis — Overexpression of pigeonpea stress-induced cold and drought regulatory gene (CcCDR) confers drought, salt, and cold tolerance in Arabidopsis — Supplementary Data 

# Overexpression of pigeonpea stress-induced cold and drought regulatory gene (*CcCDR*) confers drought, salt, and cold tolerance in *Arabidopsis*

## Supplementary Data

Data files

**Files in this Data Supplement:**

- Supplementary Data - Supplementary Data
