# Supplementary material for: Overexpression of pigeonpea stress-induced cold and drought regulatory gene (CcCDR) confers drought, salt, and cold tolerance in Arabidopsis
Source: J Exp Bot. 2014 May 27;65(17):4769–81. doi: 10.1093/jxb/eru224 (PMC4144763; doi:10.1093/jxb/eru224)
Supplement: Supplementary Data [file supp_eru224_jexbot121178_file001.pdf]

Overexpression of pigeonpea stress-induced cold and drought regulatory gene (*CcCDR*) confers osmotic and cold tolerance in Arabidopsis

Venkateswara Rao Khareedu, Srinath Tamirisa, and Dashavantha Reddy Vudem

Supplementary Data

Table S1. Selected genes and primers used for Real Time-PCR analysis.

**Gene name**	**Primer sequence**
LEA-F	GCAATCAAGAACAAGGCACA
LEA-R	TCAGTGCGAAGCCCTAAAGT
Zinc Finger (ZNF)-F	TTGCTTCTTGGAAGGCTGTT
Zinc Finger (ZNF)-R	ACCAACGGAAATAGCAGTGG
CDPK-F	TGATGAGCATTTGCAGGAAG
CDPK-R	ATCCGTCCATCCTTGTTGAG
MYB-F	GGACCAACTATCTCCGACCA
MYB-R	GTGTGTAACCGGGTCGATTC
MAPK-F	GGAGAGATGGACAGCGAGTC
MAPK-R	ACATTCCCACCAGCTTGAAC
bZIP-F	CATGTTGCTGGTTCGTCTTG
bZIP-R	CAATGGGTTTCGACGACATA
DREB2A-F	GGTGCGGAAGAGATGAAGAG
DREB2A-R	GCCAACCATAGTCTGCAACA
CBF4-F	TGGGAGGAAGAAGTTTCGTG
CBF4-R	TCAACCGTCGGAAAAGTACC
CBF1-F	GTTTGGGATGCCGACTTTGT
CBF1-R	ACCATCTCCTTCGCCCTCAT

Table S2. List of genes upregulated more than four-fold in *CcCDR* transgenic Arabidopsis subjected to drought stress.

**Gene Description**	**Genbank Accession**
Hypothetical protein	NM_125566
Legumain	NM_001203009
Defensin-like protein 16	NM_123809
Hypothetical protein	NM_113799
Plant invertase/pectin methylesterase inhibitor domain-containing protein	NM_118597
Hypothetical protein	NM_110989
Defensin-like protein 14	NM_128160
Defensin-like protein 17	NM_123810
2-oxoglutarate (2OG) and Fe(II)-dependent oxygenase-like protein	NM_129381
Pectinesterase VGDH2	NM_116082
Putative defensin-like protein 15	NM_128161
Pectinesterase PPME1	NM_105663
Hypothetical protein	NM_100326
Protein kinase family protein	NM_112945
Putative pectate lyase 19	NM_121515
Late embryogenesis abundant domain-containing protein	NM_202280
Putative calcium-binding protein CML40	NM_202478
Zinc finger (C2H2 type) family protein	NM_119735
Hypothetical protein	NM_127457
Ubiquitin-like domain-containing protein-like protein	NM_122443
Beta-fructofuranosidase, insoluble isoenzyme CWINV2	NM_001035770
Calmodulin-like protein 6	NM_116567
Hypothetical protein	NM_122752
Hypothetical protein	NM_113802
Sugar transport protein 11	NM_122233
Hypothetical protein	NM_106237
Endoglucanase 16	NM_114254
Putative clathrin assembly protein	NM_100186
Polygalacturonase 4	NM_100158
Exocyst complex component 7	NM_111787
Putative pectinesterase 50	NM_120825
Glycine rich protein 17	NM_120835
Purple acid phosphatase 23	NM_117444
Hypothetical protein	NM_113805
Plant invertase/pectin methylesterase inhibitor domain-containing protein	NM_116083

Hypothetical protein	NM_110991
Expansin A24	NM_123293
Acyl-CoA N-acyltransferases-like protein	NM_129460
Glycine rich protein 17	NM_120835
Hypothetical protein	NM_103819
Transcription factor IIS-like protein	NM_128338
Self-incompatibility S1 family protein	NM_001125236
FAD-binding and BBE domain-containing protein	NM_100078
Serine-rich protein-like protein	NM_148718
Putative membrane lipoprotein	NM_180538
Plant invertase/pectin methylesterase inhibitor domain-containing protein	NM_116458
protein ralf-like 4	NM_102592
Hypothetical protein	NM_113430
Hypothetical protein	NM_115476
Heat shock protein 22	NM_117093
Bifunctional inhibitor/lipid-transfer protein/seed storage 2S albumin-like protein	NM_105356
Hypothetical protein	NM_128902
HVA22-like protein j	NM_129161
Pectinesterase 28	NM_122669
Protein SKU5-like 11	NM_112184
Receptor-like protein kinase ANXUR2	NM_122751
bZIP family transcription factor	NM_103243
Protein SEC14-like 12	NM_119812
Hypothetical protein	NM_113430
GATA type zinc finger transcription factor-like protein	NM_001036468
RING/U-box domain-containing protein	NM_106156
Anthocyanin 5-aromatic acyltransferase 1	NM_125509
P-coumarate 3-hydroxylase	NM_106114
Hypothetical protein	NM_120781
Hypothetical protein	NM_113804
Putative protein kinase	NM_106286
NAD(P)-linked oxidoreductase-like protein	NM_104687
Hypothetical protein	NM_113819
Pectinesterase 4	NM_130271
Pleckstrin homology (PH) and lipid-binding START domain-containing protein	NM_001035785
Peroxidase 65	NM_124071
Hypothetical protein	NM_110990
Beta-1,3-glucanase 4	NM_122040
RPM1-interacting protein 4 (RIN4) family protein	NM_001036829
Leucine-rich repeat protein kinase-like protein	NM_126680

Non-specific lipid-transfer protein 4	NM_125322
Calcium-dependent protein kinase 24	NM_128707
Pectinesterase 23	NM_111563
Leucine-rich repeat protein kinase-like protein	NM_126680
Pollen Ole e 1 allergen and extensin family protein	NM_102655
Phosphoinositide 4-kinase gamma 1	NM_129648
Arabinogalactan protein 11	NM_111036
Self-incompatibility protein S1 family	NM_179311
Peroxidase 5	NM_101322
Putative pectate lyase 3	NM_101309
Trypsin inhibitor (Kunitz) domain-containing protein	NM_105888
Cellulose synthase-like protein D4	NM_119980
F-box protein	NM_128242
Plant self-incompatibility protein S1 family	NM_104003
Hypothetical protein	NM_113819
Putative polygalacturonase / pectinase	NM_124188
LOB domain-containing protein 17	NM_180039
COBRA-like protein 10	NM_112950
Putative pectate lyase 7	NM_110993
Defensin-like protein 151	NM_117245
Nuclear transcription factor Y subunit C-5	NM_124431
Hypothetical protein	NM_113803
SAUR-like auxin-responsive protein	NM_114180
Glycine-rich protein 19	NM_120837
Protein kinase family protein	NM_121655
Glycine-rich protein 16	NM_120836
Protein SEUSS-like 4	NM_125190
Self-incompatibility S1 family protein	NM_001125236
C2H2-type zinc finger protein	NM_115220
Polygalacturonase-like protein	NM_111661
Defensin-like protein 13	NM_106233
Pectinesterase 5	NM_130272
Subtilisin-like serine endopeptidase family protein	NM_105292
Germin-like protein subfamily 1 member 6	NM_111291
Cyclin D7-1	NM_120289
Adenine nucleotide alpha hydrolase domain-containing protein kinase	NM_127999
Response to low sulfur 1 protein	NM_114818
PLAC8 family protein	NM_105535
Cellulase (glycosyl hydrolase family 5) protein	NM_113521
Hypothetical protein	NM_113800
Myb domain protein 74	NM_116749

Protein kinase protein with adenine nucleotide alpha hydrolases-like domain	NM_121238
Glycine-rich protein 19	NM_120837
Cysteine/histidine-rich C1 domain-containing protein	NM_104420
Plant invertase/pectin methylesterase inhibitor domain-containing protein	NM_100953
Response to low sulfur 1 protein	NM_114818
Hypothetical protein	NM_113819
Hypothetical protein	NM_116129
Nodulin MtN21 /EamA-like transporter family protein	NM_124120
SKU5 similar 14	NM_104432
Pectin lyase-like protein	NM_127951
Protein exordium like 6	NM_111167
Calcium-dependent lipid-binding domain-containing protein	NM_100333
Defensin-like protein 103	NM_179669
Copper transporter 3	NM_125294
Non-specific lipid-transfer protein 12	NM_115018
GDSL esterase/lipase 1	NM_123464
Hypothetical protein	NM_124417
ABC transporter G family member 9	NM_118876
Hypothetical protein	NM_106347
Endoglucanase 13	NM_130019
Jasmonate-regulated protein	NM_115455
Hypothetical protein	NM_123351
Putative caffeoyl-CoA O-methyltransferase	NM_105469
LOB domain-containing protein 2	NM_100510
Cysteine/histidine-rich C1 domain-containing protein	
Profilin 5	NM_127535
Cytochrome P450, family 86, subfamily C, polypeptide 3	NM_001198050
Ralf-like 8 protein	NM_104837
Actin depolymerizing factor 7	NM_118691
Phosphoinositide phospholipase C 6	NM_179996
Hypothetical protein	NM_104594
MLP-like protein 168	NM_103228
Hypothetical protein	NM_116737
Phosphate starvation-induced protein 2	NM_105959
Calcium-dependent lipid-binding domain-containing protein	NM_100333
Hypothetical protein	NM_115476
Hypothetical protein	NM_100607
Protein kinase domain-containing protein	NM_111149
Sphingolipid delta-4 desaturase	NM_116731
NAC domain-containing protein 12	NM_103011
Protein kinase protein with adenine nucleotide alpha hydrolases-like domain	NM_121238

Syntaxin 1B/2/3	NM_111251
Fasciclin-like arabinogalactan protein 17	NM_120722
Hypothetical protein	NM_102604
Ethylene response factor PUCHI	NM_121861
Putative pectinesterase 48	NM_120823
Mitogen-activated protein kinase kinase kinase 18	NM_100389
Beta-galactosidase 11	NM_119667
Carbohydrate-binding X8 domain-containing protein	NM_105358
Self-incompatibility protein S1 family	NM_179311
Hypothetical protein	NM_113796
Hypothetical protein	NM_105561
Core-2/I-branching beta-1,6-N-acetylglucosaminyltransferase family protein	
Ankyrin repeat-containing protein	NM_180494
Hypothetical protein	NM_102764
Plant invertase/pectin methylesterase inhibitor domain-containing protein	NM_130273
Putative pectinesterase 67	NM_112582
Alpha/beta-hydrolases family protein	NM_115284
Hypothetical protein	NM_113800
Serine protease inhibitor / potato inhibitor I domain-containing protein	NM_114553
Phytosulfokine-beta	NM_119934
Auxin-responsive GH3 family protein	NM_121341
ATP binding microtubule motor family protein	NM_123614
Protein SKU5 similar 12	NM_104433
Putative defensin-like protein 84	NM_001037000
Hypothetical protein	NM_102435
RAB GDP-dissociation inhibitor	NM_120992
Aspartic-type endopeptidase	NM_105580
Defensin-like protein 106	NM_001036141
Roline-rich extensin-like receptor kinase 4	NM_127403
Cation/H+ exchanger 6A	NM_179280
High affinity nitrate transporter 2.6	NM_114375
Carbonic anhydrase	NM_100683
Phosphate starvation-induced protein 2	NM_105959
Leucine-rich repeat receptor-like protein kinase	NM_148476
Heat stress transcription factor A-6a	NM_123751
Hypothetical protein	NM_124789
Thioredoxin-like 1-3	NM_128887
Plant invertase/pectin methylesterase inhibitor domain-containing protein	NM_179746
Cytochrome P450, family 703, subfamily A, polypeptide 2	NM_100010
Calcium-binding EF hand family protein	NM_118917
Self-incompatibility S1 family protein	NM_122507

Cytochrome P450, family 96, subfamily A, polypeptide 15	NM_104570
RING/U-box domain-containing protein	NM_202179
Calcium-dependent protein kinase 14	NM_201932
Hypothetical protein	NM_114814
Hypothetical protein	NM_113796
Putative phospholipid:diacylglycerol acyltransferase 2	NM_114352
Phosphate transporter PHO1-9	NM_113828
Protein SKU5 similar 12	NM_104433
Hydroxyproline-rich glycoprotein-like protein	NM_127786
Mto 1 responding down 1 protein	NM_104226
Pectinesterase 21	NM_111434
Microspore-specific promoter 2	NM_124050
Arabinogalactan protein 23	NM_115629
Protein SKU5-like 13	NM_112185
Receptor like protein 15	NM_106079
TPX2 (targeting protein for Xklp2) family protein	NM_147942
Protein RALF-like 15	NM_001084470
C2 domain-containing protein	NM_126924
DNA binding protein	NM_001125551
VQ motif-containing protein	NM_119933
ECA1 gametogenesis related family protein	NM_001085117
Glutathione S-transferase	NM_100175
Phosphate starvation-induced protein 2	NM_105959
Hypothetical protein	NM_125730
Hypothetical protein	NM_101756
Hypothetical protein	NM_101090
Mitogen-activated protein kinase kinase kinase 19	NM_126108
Pectin lyase-like protein	NM_127951
Octicosapeptide/Phox/Bem1p domain-containing protein	NM_124370
NDR1/HIN1-like 25	NM_123055
Hypothetical protein	NM_119892
Mitogen-activated protein kinase kinase kinase 19	NM_126108
Hypothetical protein	
Fasciclin-like arabinogalactan family protein	NM_180541
CBL-interacting serine/threonine-protein kinase 13	NM_128969
Arabinogalactan protein 6	NM_121442
Hypothetical protein	NM_129077
Hypothetical protein	NM_117533
Glycosyl hydrolase-17	NM_129520
VQ motif-containing protein	NM_119933
RHOMBOID-like protein 5	NM_104136

C2H2-like zinc finger protein	NM_119694
Fasciclin-like arabinogalactan protein 3	NM_128006
Glycosyl hydrolase-like protein	NM_116136
Protein kinase protein with adenine nucleotide alpha hydrolases-like domain	NM_121238
Hydroxyproline-rich glycoprotein-like protein	NM_127786
Purple acid phosphatase 6	NM_104516
Beta-1,3-N-Acetylglucosaminyltransferase family protein	NM_148388
Hypothetical protein	NM_127797
Hypothetical protein	NM_104484
Callose synthase 5	NM_179622
Protein SKU5-like 13	NM_112185
Hypothetical protein	NM_202502
Hypothetical protein	NM_116103
EID1-like F-box protein 3	NM_116171
Plant invertase/pectin methylesterase inhibitor domain-containing protein	NM_124382
Cellulose synthase-like protein D1	NM_128870
Hypothetical protein	NM_124052
HSP20-like chaperone	NM_104232
Response to low sulfur 3 protein	NM_114817
Hypothetical protein	NM_123143
Non-specific lipid-transfer protein 3	NM_125323
Hypothetical protein	NM_128285
Homeobox-leucine zipper family protein	NM_001198174
MYB-like 102	NM_118264
Cation/H(+) antiporter 23	NM_100438
Putative beta-1,3-glucanase	NM_122046
Self-incompatibility S1 family protein	NM_121244
EID1-like F-box protein 3	NM_116171
F-box protein	NM_120483
Peroxidase 4	NM_101321
2-oxoglutarate-Fe(II)-dependent oxygenase domain-containing protein	NM_114822
Jasmonic acid carboxyl methyltransferase	NM_101820
Carbonic anhydrase	NM_128381
Plant invertase/pectin methylesterase inhibitor domain-containing protein	NM_102184
Beta-xylosidase 3	NM_121010
Plant invertase/pectin methylesterase inhibitor domain-containing protein	NM_130302
Hypothetical protein	NM_001084980
ECA1-like gametogenesis related family protein	NM_001084617
Ethylene-responsive transcription factor ERF020	NM_105820
Germin-like protein subfamily 2 member 3	NM_111467
Ethylene-responsive transcription factor ERF016	NM_147879

HXXXD-type acyl-transferase-like protein	NM_100232
Hypothetical protein	NM_203145
Plant invertase/pectin methylesterase inhibitor domain-containing protein	NM_001035569
Ralf-like 9 protein	NM_104838
ABC transporter A family member 11	NM_125563
Carboxypeptidase D	NM_115060
Stigma-specific stig1-like protein	NM_148459
Arabidopsis thaliana dehydration-responsive element-binding protein CBF1	NM_118681
Hypothetical protein	NM_124461
WD40 domain-containing protein	NM_103834
Putative F-box protein	NM_111916
Auxin-responsive protein IAA31	NM_112640
Hypothetical protein	NM_148506
Germin-like protein subfamily 2 member 3	NM_111467
Stigma-specific stig1-like protein	NM_148459
C2H2-type zinc finger protein	NM_111882
F-box protein	NM_116088
Heat shock protein 18.2	NM_125364
Hypothetical protein	NM_130099
Hypothetical protein	NM_125670
Late embryogenesis abundant protein, group 6	NM_127875
Pectinesterase 4	NM_130271
Protein TAPETUM 1	NM_114164
Thioesterase family protein	NM_103226
Cytochrome P450, family 704, subfamily B, polypeptide 1	NM_105617
Expansin B5	NM_115921
Rossmann-fold NAD(P)-binding domain-containing protein	NM_104019
Hypothetical protein	NM_123989
Putative F-box protein	NM_106437
Hypothetical protein	NM_148501
Putative defensin-like protein 70	NM_001036997
Putative galacturonosyltransferase-like 4	NM_111501
Hypothetical protein	NM_001125311
Hypothetical protein	NM_201845
Expansin A25	NM_123292
Hypothetical protein	NM_148430
Putative pectate lyase 6	NM_126327
Protein phosphatase	NM_125312
Glutathione S-transferase-like protein	NM_101813
CBL-interacting serine/threonine-protein kinase 18	NM_102663
Defensin-like protein 117	NM_001036127

Putative S-acyltransferase	NM_120589
Putative germin-like protein subfamily 2 member 5	NM_180544
Protein exordium like 6	NM_111167
Hypothetical protein	NM_001084987
Hypothetical protein	NM_101882
Transcription factor MYB75	NM_104541
Tryptophan-rich sensory protein-like protein	NM_130344
Putative inorganic phosphate transporter 1-6	NM_123700
Arabidopsis thaliana dehydration-responsive element-binding protein CBF4	NM_124578
UDP-glucosyl transferase 73C	NM_129232
Pectinesterase inhibitor 1	NM_103698
Calmodulin-binding protein-like protein	NM_115710
Phosphatidic acid phosphatase (PAP2) family protein	NM_112706
Hypothetical protein	NM_114179
C2H2-type zinc finger-containing protein	NM_111147
Nitrate transporter 1.8	NM_118288
Putative protein phosphatase 2C 71	NM_122403
Hypothetical protein	NM_120604
Cation/H(+) antiporter 5	NM_100690
BTB/POZ/Kelch-associated protein	NM_116346
Hypothetical protein	NM_120604
Putative glucan endo-1,3-beta-glucosidase A6	NM_117483
14-3-3-like protein GF14 iota	NM_102411
TRAF-like family protein	NM_126455
Subtilisin-like serine endopeptidase family protein	NM_125272
Putative membrane lipoprotein	NM_180539
Hypothetical protein	NM_118063
1-aminocyclopropane-1-carboxylate synthase 2	NM_100030
Hypothetical protein	NM_202323
Lipase class 3 family protein	NM_117784
Hypothetical protein	NM_102708
Putative clathrin assembly protein	NM_102333
Actin depolymerizing factor 10	NM_124615
Ethylene-responsive transcription factor ERF115	NM_120813
Major facilitator protein	NM_127415
Hypothetical protein	NM_104790
Hypothetical protein	NM_101882
S-phase kinase-associated protein 1	NM_113463
Inositol oxygenase 4	NM_001203915
Monogalactosyldiacylglycerol synthase 3	NM_001124829
Putative protein phosphatase 2C 71	NM_122403

ChaC-like family protein	NM_122523
Protein kinase protein with adenine nucleotide alpha hydrolases-like domain	NM_101616
Protein kinase family protein	NM_112767
Oxidative stress 3	NM_125038
Pollen_Ole_e_I-domain containing protein	NM_123956
Pectinesterase 20	NM_130323
Arabidopsis thaliana dehydration-responsive element binding protein DREB2A 2A	NM_001036760
DEDDy 3'-5' exonuclease domain-containing protein	NM_112078
Subtilase family protein	NM_125373
F-box/kelch-repeat protein	NM_111338
Non-specific lipid-transfer protein 3	NM_125323
LOB domain-containing protein 33	NM_120690
3-ketoacyl-CoA synthase 7	NM_105785
Dehydrodolichyl diphosphate synthase 8	NM_125444
Tryptophan-rich sensory protein-like protein	NM_130344
Hypothetical protein	NM_001085016
Glycine-rich protein 20	NM_120838
Protein kinase family protein	NM_121896
Hypothetical protein	NM_001123865
Cellulose synthase-like protein G2	NM_118532
TIR-NBS class of disease resistance protein	NM_105280
Putative mannan synthase 15	NM_117415

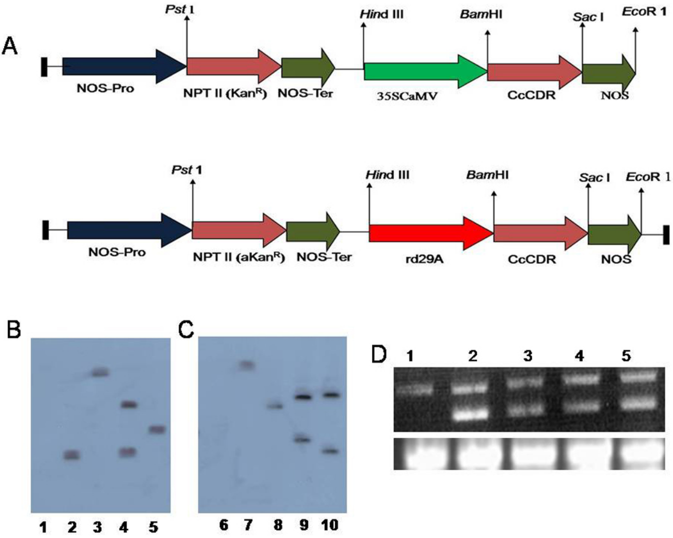

Supplemental Figure 1. Molecular analysis of *CcCDR*-transgenic *Arabidopsis* plants.

A. Restriction map of T-DNA region of pBI121 containing *CcCDR* expression units with CaMV35S and rd29A promoters.

B and C. Southern blot analysis of CaMV35S-*CcCDR* transformants (2,3,4,5) and rd29A-*CcCDR* plants (7,8,9,10). 1 and 6 are wild-type plants.

D. RT PCR analysis showing the expression of CcCDR in transgenic *Arabidopsis*. Lane 1: wild-type; Lanes 2 & 3: 35S transgenic lines; Lanes 4 & 5: rd29A transgenic subjected to 200 mM mannitol stress.

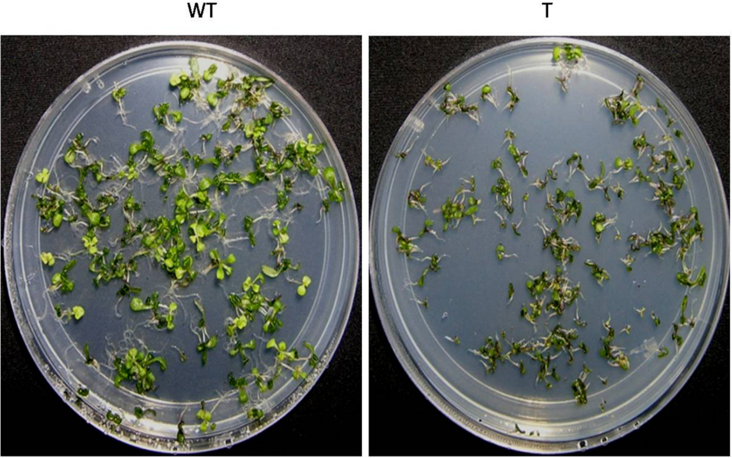

Supplemental Figure 2. Twenty-day-old WT and transgenic (T) *Arabidopsis* seedlings grown on MS medium containing 1.5 μM ABA.
